# The Relationship Between Empowering Motivational Climate in Physical Education and Social Responsibility of High School Students: Chain Mediating Effect Test

**DOI:** 10.3389/fpsyg.2021.752702

**Published:** 2022-01-12

**Authors:** Ke-lei Guo, Qi-shuai Ma, Shu-jun Yao, Chao Liu, Zhen Hui

**Affiliations:** ^1^School of Physical Education and Health, Zhaoqing University, Zhaoqing, China; ^2^School of Physical Education, Huaibei Normal University, Huaibei, China; ^3^School of Marxism, Zhaoqing University, Zhaoqing, China

**Keywords:** high school students, empowering motivational climate, social responsibility, interpersonal disturbance, general self-efficacy

## Abstract

**Objective:** This study aimed to contribute to understanding the mechanisms underlying the association between empowering motivational climate in physical education and social responsibility among high school students, and have important implications for interventions aimed at improving social responsibility among high school students.

**Methods:** Through the quota sampling, 802 students (average age = 17 years, *SD* = 0.97 years) that complied with the requirements were surveyed from Anhui Province in China. Empowering motivational climate in physical education, social responsibility, interpersonal disturbance, and general self-efficacy were assessed using standard scales. For data analysis, Pearson’s correlation analysis, structural equation model test, and bias-corrected percentile Bootstrap method were carried out in turn.

**Results:** (1) Common method biases can be accepted in this study, and the correlation among empowering motivational climate in physical education, social responsibility, interpersonal disturbance, and general self-efficacy are all significant; (2)Empowering motivational climate in physical education, interpersonal disturbance and general self-efficacy can all predict social responsibility significantly; (3) Structural equation modeling indicates good fit: χ^2^/*df* = 2.86, RMESA = 0.068, CFI = 0.92, NNFI = 0.91, NFI = 0.90, GFI = 0.93. It indicates that interpersonal disturbance and general self-efficacy can play mediating roles between empowering motivational climate in physical education and social responsibility, respectively. After that, interpersonal disturbance as well as general self-efficacy in turn plays the chain mediating effect in the relationship between empowering motivational climate in physical education and social responsibility. The effect size of the mediating effect of interpersonal disturbance and general self-efficacy in the relationship between empowering motivational climate in physical education and social responsibility is 0.048 and 0.148, respectively, and the effect size of the chain mediating effect is 0.031.

**Conclusion:** Empowering motivational climate in physical education not only has a direct effect on social responsibility among high school students, but also influences social responsibility by the chain mediating effect of interpersonal disturbance and general self-efficacy.

## Introduction

Recently, Duda (2013) and Appleton et al. (2016) suggested a hierarchical and multidimensional conceptualization of the coach-created motivational climate that integrates the major social environmental dimensions emphasized within achievement goal theory (AGT) (Nicholls, 1989; Ames, 1992) and self-determination theory (SDT) (Deci and Ryan, 1985, 2000). AGT proposes that the motivational climate is the social environment surrounding pupils and is a function of what teachers say and do, how they organize, communicate, try to motivate, and use praise and feedback following desirable performance or mistakes (Duda, 2001). Duda suggests that the motivational climate created is multidimensional and can be more or less empowering (i.e., those which are more task involving, autonomy supportive, and socially supportive) and disempowering (i.e., they are more ego involving and controlling/relatedness thwarting) climates (Milton et al., 2018). The motivational climate created by teachers in physical education has received considerable attention in previous research and holds important pedagogical implications for students’ motivation (Braithwaite et al., 2011), and psychological responses in physical education (Van den Berghe et al., 2014).

Social responsibility is the essential quality of every citizen in today’s society. In October 2019, China issued the “Implementation Outline of Civic Morality construction in the New Era,” which clearly requires that the establishment of morality and the cultivation of people should run through the whole process of school education and guide young students to enhance their sense of social responsibility. In the “High School Physical Education and Health Curriculum Standards (2017 edition),” social responsibility is also regarded as an important part of the physical literacy. High school stage is a critical period for the formation of students’ world outlook, values and outlook on life. As the successors of national construction, high school students’ social responsibility is directly related to the success or failure of national construction. However, due to negative influences such as bad behaviors and thoughts, contemporary teenagers’ social responsibility is facing serious challenges (Lan et al., 2015). Physical education is an important way to cultivate teenagers’ social responsibility, and the teacher-created empowering motivational climate have a beneficial effect on the moral development of high school students (Pengyu and Jianing, 2020). Exploring the relationship between empowering motivational climate in physical education and social responsibility is an important way to cultivate teenagers’ social responsibility. In recent years, the research on the relationship between the two has attracted more and more attention in the field of physical education psychology (Liwei and Zhixiong, 2019). Based on this, this study aimed to contribute to understanding the mechanisms underlying the association between empowering motivational climate in physical education and social responsibility among high school students, and have important implications for interventions aimed at improving social responsibility among high school students.

### The Relationship Between Empowering Motivational Climate in Physical Education and Social Responsibility

Social responsibility refers to the correct evaluation and cognition of social individuals to their responsibilities in social public life, which is reflected in their individual psychological qualities in their behaviors and emotions, mainly including three dimensions of social responsibility cognition, social responsibility emotion, and social responsibility behavior (Yong and Xiaohong, 2008). Previous studies have explored the influencing factors of students’ social responsibility mainly from the perspectives of social service experience, interpersonal relationship, achievement motivation, family function, etc., among which, the empowering motivational climate in physical education is unanimously considered to be an important and favorable factor to cultivate students’ social responsibility (Pengyu and Jianing, 2020). According to the theory empowering and disempowering motivational climate proposed by Duda (2013), work involvement in achievement goal theory and social support and autonomous support in self-determination theory are important foundations of empowering motivation climate, and the empowering motivational climate created by teachers in physical education plays an important role in students’ moral development. The Teaching Personal and Social Responsibility Model (TPSR) (Hellison and Wright, 2003) helped teachers to structure classes and promoted the learning of responsible student behaviors, the TPSR improved not only personal and social responsibility but also prosocial behavior. A large number of studies have shown that the social support provided by teachers plays an extremely important role in enhancing students’ social responsibility (Demaray and Malecki, 2002; Ruimiao, 2012; Manzano-Sánchez et al., 2021). Canonical correlation showed that students’ perceptions of an empowering motivational climate were related with positive social behavior (Kolovelonis et al., 2015; González-Valero et al., 2019). Therefore, hypothesis 1 is proposed: empowering motivation climate in physical education can positively predict high school students’ social responsibility.

### Mediating Role of Interpersonal Disturbance

Interpersonal disturbance is proposed on the basis of theories related to interpersonal relationship. It refers to the inharmonious interpersonal relationship caused by various reasons in the process of interpersonal communication, accompanied by negative emotions such as loneliness and anxiety (Richang, 1999). Lim and Wang (2009) pointed out that the core context provided by physical education teachers is social environment factors, that is, when physical education teachers provide sufficient support, students’ satisfaction of autonomy, sense of competence and sense of relationship will further generate more positive behaviors through motivation forms. These results suggest that interpersonal disturbance may be a mediating variable between the empowering motivational climate and social responsibility. First of all, according to the theory of empowering motivation climate, in physical education teaching, the motivational climate created by teachers has a very important influence on students’ psychological reactions (such as interpersonal relationship) in physical activities (Duda, 2013). Secondly, studies have shown that interpersonal relationship is considered as an important predictor of social responsibility among many factors (Ming and Jinang, 2010; Wray-Lake and Syvertsen, 2011). Huang et al. (2016) found that interpersonal relationship has a significant positive predictive effect on social responsibility. Other studies have shown that there is an indirect and complex relationship between empowering motivation climate and social responsibility (Metzler, 2017), and the role of empowering motivation climate on social responsibility is influenced by other factors (Ntoumanis, 2001). Accordingly, hypothesis 2 is put forward: interpersonal disturbance plays an intermediary role between the empowering motivational climate and social responsibility.

### The Mediating Role of General Self-Efficacy

General self-efficacy refers to an individual’s confidence in whether he can use his skills to complete a certain work behavior (Bandura, 1999). Related studies have found that there is a significant positive correlation between general self-efficacy and adolescents’ social responsibility, and the higher the general self-efficacy, the higher the social responsibility (Ning and Chaoyi, 2015). General self-efficacy can have a positive impact on individual attitudes and behaviors, while social responsibility is actually an individual’s positive attitude and behavioral tendency, and general self-efficacy is an important endogenous factor influencing adolescents’ social responsibility (Baoling and Jiliang, 2015). Research shows that teachers’ teaching style can effectively predict students’ self-efficacy and interest in learning, and teachers’ teaching style is significantly related to students’ academic self-efficacy (Yue, 2012). Teachers’ love for students can enhance students’ sense of self-efficacy (Guixue, 2013). Other studies show that teacher evaluations also have an impact on students’ self-efficacy. Teachers’ positive evaluations of students can promote students to successfully complete their learning tasks and improve their self-confidence, which is conducive to promoting students’ learning self-efficacy (Hao, 2011). A study shows that when physical education teachers provide a psychological environment with empowerment characteristics, students’ satisfaction with autonomy, self-efficacy and sense of relationship can further generate more positive behaviors through motivational forms (Deci and Ryan, 1985, 2000). Other studies have proved that external environmental conditions may indirectly affect the physical and mental characteristics of individuals through general self-efficacy (Jianwen and Tao, 2007). Self-efficacy mediated the relationship between transformational leadership style of teachers and social responsibility of junior high school students (Yingqi, 2018), and self-efficacy mediated the relationship between social support and social responsibility of medical students (Hongmei et al., 2018). Therefore, hypothesis 3 is proposed: general self-efficacy plays a mediating role between the empowering motivational climate and social responsibility.

### The Chain Mediating Effect of Interpersonal Disturbance and General Self-Efficacy

As for the two mediating variables of interpersonal disturbance and general self-efficacy in this study, studies have shown that interpersonal relationship has a significant positive predictive effect on general self-efficacy (Dingding et al., 2018), and interpersonal disturbance has a significant negative predictive effect on emotional regulation self-efficacy (Qian and Shou-cai, 2009; Jianyu and Xiaohua, 2016), interpersonal disturbance is negatively correlated with general self-efficacy (Yanyan et al., 2015). Individuals with interpersonal disturbance have certain obstacles in making friends, communicating and talking, which are likely to cause problems such as adaptation difficulties (Yongzhi and Xiaoli, 2014). However, general self-efficacy refers to the confidence and subjective judgment of an individual’s ability to implement a certain behavioral goal, which plays a positive role in the individual’s learning ability and moral development (Wenfeng and Fuming, 2004). Therefore, they are in a state of antagonism. The teacher-created positive empowering motivational climate can reduce the interpersonal disturbance of adolescents, improve the individual self-efficacy, and then enhance their social responsibility. Therefore, hypothesis 4 is proposed: interpersonal disturbance and general self-efficacy play a chain mediating role between the empowering motivational climate in physical education and social responsibility.

**FIGURE 1:**
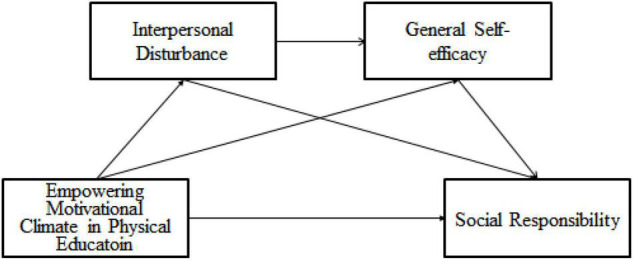
Conceptual framework.

To sum up, in order to investigate the relationship between the empowering motivational climate in physical education and social responsibility, this study intends to build a chain mediation model (as shown in Figure 1) and verify the following aspects: (1) Empowering motivational climate in physical education significantly negatively predicted high school students’ social responsibility; (2) Interpersonal disturbance and general self-efficacy played a separate mediating role between the empowering motivational climate in physical education and social responsibility; (3) Interpersonal disturbance and general self-efficacy played a chain mediating role between the empowering motivational climate in physical education and social responsibility.

## Materials and Methods

### Procedure and Participants

Using stratified cluster sampling, 1 high school was selected from urban and rural areas in the three regions of southern, central and northern Anhui Province, and 2 classes were randomly selected from each high school and each grade. Questionnaires will be issued during class and collected on the spot. A total of 935 people from 36 classes were distributed questionnaires. After eliminating invalid questionnaires caused by lack of regular answering data and other reasons, 802 valid questionnaires were collected. The average age of the participants was 17.00 ± 0.972, including 369 males and 433 females. There are 312 students in the first grade, 277 students in the second grade, and 213 students in the third grade. The study was in accordance with the Declaration of Helsinki, and was approved by the Institutional Review Board of the School of Physical Education and Health at Zhaoqing University of China, and all participants signed an informed consent form and were paid for their participation.

### Measures and Instruments

The empowering motivational climate was evaluated by the empowering motivational climate questionnaire in physical education (EMCQ-PE), which is a sub-questionnaire of the Empowering and Disempowering Motivational Climate Questionnaire in physical education (EDMCQ-PE), and was modified by Pengyu and Jianing (2020) based on Duda (2013)’s Empowering Motivational Climate Questionnaire in physical education (EMCQ-PE). The questionnaire has one dimension, including 17 questions (for example: “in physical education class, teachers encourage students to cooperate fully in class.”). Likert’s 7-point evaluation is used, where 1 represents strongly disagree and 7 represents strongly agree. The higher the score, the higher the level of empowering motivational climate felt by students. This questionnaire was used by Pengyu and Jianing (2020) and its reliability was tested. The internal consistency coefficient α of the questionnaire was 0.92. In this study, the internal consistency coefficient α of the questionnaire was 0.89.

The social responsibility was assessed by the Middle School Students’ Social Responsibility Questionnaire (MSSSRQ), which is based on Lixue’s middle school students’ social responsibility questionnaire (Xue, 2004). The MSSSRQ is a five-point Likert scale with 35 items and three dimensions: social responsibility cognition, social responsibility emotion, and social responsibility behavior. Each item is scored from 1 (completely inconsistent) to 5 (completely consistent), and the total score has a range of 35–175, and a higher score shows a higher level of social responsibility. Both exploratory and confirmatory factor analyses have supported the construct validity of the three dimensions. The internal consistency coefficient of the questionnaire is between 0.49∼0.85, and the splitter reliability coefficient is between 0.46∼0.81 (Xue, 2004). Moreover, a previous study proved that this scale was fairly in a sample of high school students (Qiang, 2011). In this study, the Cronbach’s α of the total scale was 0.74, and the Cronbach’s α of each sub-scale is 0.71∼0.76.

The interpersonal disturbance was assessed by the Interpersonal Relationship Assessment Scale (IRAS), which is based on Zheng Richang’s the interpersonal relationship assessment scale (Richang, 1999). The IRAS is a five-point Likert scale with 28 items and four dimensions: heterosexual communication problems, trouble of making friends, communication and conversation problems, and trouble in dealing with people and things. A “yes” score 1, a “no” score 0; The higher the score, the higher the degree of interpersonal distress. The scores of 0∼8, 9∼14, and 15∼28, respectively, represent the three grades of good, medium, and serious interpersonal relationships. Both exploratory and confirmatory factor analyses have supported the construct validity of the four dimensions. The Cronbach’s α of the total scale was 0.86, and the Cronbach’s α of each sub-scale is 0.76∼0.89. Additionally, a previous study demonstrated that this scale was conducted well in a sample of high school students (Yawen et al., 2020). In this study, the Cronbach’s α of the total scale was 0. 85, and the Cronbach’s α of each sub-scale is 0.72∼0.77.

The general self-efficacy was evaluated by the General Self-Efficacy Scale (GSES) (Schwarzer et al., 1999), which was modified based on Wang Caikang’s general self-Efficacy scale (Caikang et al., 2001). The GSES is a four-point Likert scale and comprises 10 items. Each item is valued from 1 (completely inconsistent) to 4 (completely consistent). The total score can be from 10 to 40, and a higher score shows a higher level of general self-efficacy. The internal consistency coefficient of GSES is 0.87 (Caikang et al., 2001), Moreover, a previous study demonstrated that this scale was conducted well in a sample of high school students (Rui et al., 2017). The Cronbach’s α in this study was 0.90.

### Procedure and Statistical Analysis

Taking the class as the unit, the whole group test was conducted. The main examiners were the post-graduates majoring in physical education who had received professional training. The test was conducted with the consent of the school leaders, the head teacher and the subjects themselves. The collective test was adopted, and the principles of anonymity, confidentiality, and voluntary filling were emphasized. The questionnaire was distributed and collected on the spot.

SPSS 21.0 and Amos 20.0 were used to analyze the data. The internal consistency test and Pearson correlation coefficient analysis were carried out for interpersonal disturbance, general self-efficacy, empowering motivational climate, and social responsibility of high school students. Amos was used to test the degree of fit between the measured model and the data actually collected, and the bias correction Bootstrap method was used to test the mediation effect.

## Results

### Common Method Deviation Test

In this study, only self-reported data was collected, which may cause common method bias (Podsakoff et al., 2003). According to the suggestions of Hao and Lirong (2004), necessary controls have been carried out in the process of measurement, such as protecting the anonymity of responders and using reverse expressions for some items. In order to further improve the rigor of the study, the Harman single factor test was used to test the deviation of the common method before data analysis. The results showed that there were seven factors with eigenvalues greater than 1, explaining 63. 64% of the variation, and the variance explained by the first factor was 22. 86%, far less than the critical value of 40% (Podsakoff et al., 2003). Therefore, there is no serious common method bias in this study.

### Descriptive Statistical and Correlation Analysis

As shown in Table 1, the correlation coefficients of empowering motivational climate in physical education, interpersonal disturbance, general self-efficacy, and social responsibility were all statistically significant. Among them, there was a significant positive correlation between motivation climate, general self-efficacy, and social responsibility (*P* < 0.01), and a significant negative correlation with interpersonal disturbance (*P* < 0.01).

**TABLE 1:** Mean, standard deviation, and correlation coefficient of each variable.

	*M*	*SD*	1	2	3	4
1. General self-efficacy	2.724	0.686	1			
2. Interpersonal disturbance	1.298	0.236	−0.273**	1		
3. Social responsibility	3.917	0.499	0.447**	−0.276**	1	
4. Empowering motivational climate in physical education	5.887	0.858	0.287**	−0.222**	0.498**	1

*N = 802.*

* p < 0.05; ** p < 0.01.

As shown in Tables 2, 3. Female empowering motivational climate in physical education score is significantly higher than male. Male interpersonal disturbance score is significantly higher than female. Male general self-efficacy score is significantly higher than female. Female social responsibility score is significantly higher than male. There was no significant difference in empowering motivational climate in physical education among different grades. Grade one students interpersonal disturbance score is significantly higher than grade three students. There was no significant difference in general self-efficacy among different grades. Grade three students social responsibility score is significantly higher than grade one students.

**TABLE 2:** Differences in gender.

	Gender	Number	*M* ± *SD*	*t*	*p*
Empowering motivational climate in physical education	male	369	5.75 ± 0.83	5.79	0.000
	female	433	5.92 ± 0.91		
Interpersonal disturbance	male	369	1.32 ± 0.24	4.98	0.000
	female	433	1.27 ± 0.22		
General self-efficacy	male	369	2.82 ± 0.68	5.17	0.000
	female	433	2.51 ± 0.67		
Social responsibility	male	369	3.82 ± 0.50	3.12	0.000
	female	433	3.98 ± 0.48		

*N = 802.*

* p < 0.05; ** p < 0.01.

**TABLE 3:** Differences in grade.

	Grade	Number	*A* *g* *e*	*M* ± *SD*	*F*	*p*
Empowering motivational climate in physical education	One	312	16.01 ± 0.84	4.82 ± 0.73	2.23	0.174
	Two	277	16.91 ± 0.98	5.88 ± 0.89		
	Three	213	18.12 ± 1.03	6.04 ± 0.95		
Interpersonal disturbance	One	312	16.01 ± 0.84	1.32 ± 0.24	11.16	0.000
	Two	277	16.91 ± 0.98	1.25 ± 0.22		
	Three	213	18.12 ± 1.03	1.17 ± 0.19		
General self-efficacy	One	312	16.01 ± 0.84	2.75 ± 0.69	1.64	0.454
	Two	277	16.91 ± 0.98	2.81 ± 0.65		
	Three	213	18.12 ± 1.03	2.56 ± 0.64		
Social responsibility	One	312	16.01 ± 0.84	3.21 ± 0.45	8.76	0.000
	Two	277	16.91 ± 0.98	3.75 ± 0.51		
	Three	213	18.12 ± 1.03	3.95 ± 0.55		

*N = 802.*

* p < 0.05; ** p < 0.01.

### Construction and Test of Structural Equation Model

Amos 21.0 was used to test the fitting degree between the measurement model and the actual data. The results show that, χ^2^/*df* = 2.86, RMESA = 0.068, CFI = 0.92, NNFI = 0.91, NFI = 0.90, GFI = 0.93. In the measurement model, as shown in Table 4, the standardized factor loads of all the observed variables ranged from 0.59 to 0.82, and the compose reliability (CR) of the four exogenous structural variables ranged from 0.65 to 0.87, indicating that the scale reliability was measured stably. The analysis of variance (AVE) of the four structural variables ranged from 0.62 to 0.79. Each index reached the ideal standard of the measurement model. It shows that the measurement model meets the ideal standard, and the structural model can be further tested. The reliability analysis of each scale is shown in Table 5.

**TABLE 4:** AVE and CR of dimensions.

Dimensions naming	AVE	CR
Empowering motivational climate questionnaire in physical education	0.75	0.85
Heterosexual communication problems	0.79	0.82
Trouble of making friends	0.66	0.65
Communication and conversation problems	0.74	0.79
Trouble in dealing with people and things	0.76	0.81
General self-efficacy	0.62	0.75
Social responsibility cognition	0.78	0.87
Social responsibility emotion	0.65	0.71
Social responsibility behavior	0.75	0.83

**TABLE 5:** Reliability analysis of scales.

Scale	Factor naming	Cronbach’s α coefficient
EMCQ-PE	Empowering motivational climate questionnaire in physical education	0.89
IRAS	Heterosexual communication problems	0.72
	Trouble of making friends	0.76
	Communication and conversation problems	0.75
	Trouble in dealing with people and things	0.77
GSES	General self-efficacy	0.90
MSSSRQ	Social responsibility cognition	0.76
	Social responsibility emotion	0.71
	Social responsibility behavior	0.75

*N = 802.*

* p < 0.05; ** p < 0.01.

If the correlation between independent variables is very high, there may be a multicollinearity problem. According to the formula VIFj = 1/(1−*R*_*j*_^2^) (*R*_*j*_^2^ is the measurement coefficient obtained by regression of independent variables to other independent variables; variance inflation factor, VIF). Through calculation, the VIF of all predictive variables in this study is not higher than 5, so it can be considered that there is no multicollinearity problem (Zhonglin et al., 2018).

**FIGURE 2:**
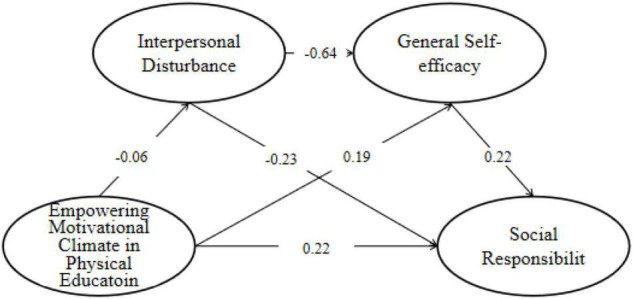
Chain-mediated model of physical education empowerment motivation climate affecting social responsibility (*n* = 802).

This study first tested the direct predictive effect of the climate of empowering motivation climate in physical education on social responsibility. The results show that the model fits the data well: χ^2^/*df* = 2.79, RMSEA = 0.09, NFI = 0.91, GFI = 0.91, CFI = 0.93. The empowering motivational climate in physical education can significantly positively predict social responsibility (β = 0.289, *P* < 0.01), and hypothesis 1 was verified. This study then constructed a chain mediation model of interpersonal disturbance and general self-efficacy, and the results showed that the mediation model of interpersonal disturbance and general self-efficacy fitted well (see Table 6 and Figure 2).

**TABLE 6:** Fitting index of the mediating role model of interpersonal disturbance and general self-efficacy.

χ ^2^/df	CFI	NFI	GFI	NNFI	RMESA
2.79	0.93	0.91	0.91	0.92	0.093

*N = 802.*

* p < 0.05; ** p < 0.01.

### Significance Test of Mediating Effect

The bias corrected bootstrap was used to test the mediating effect of interpersonal distress and general self-efficacy between the empowerment motivation climate of physical education and social responsibility (Jie and MinQiang, 2012). A total of 5000 bootstrap samples were randomly selected from the original samples (*n* = 802) to estimate the indirect effect.

Table 7 shows the standardized estimates of each indirect path and the 95% confidence interval of the mediation effect. If the 95% confidence interval does not contain 0, the mediation effect is significant (Jie et al., 2012). According to Table 7, the 95% confidence intervals of the three indirect paths do not include 0, indicating that the mediating effect of interpersonal distress is significant (the mediating effect value is 0.01); The mediating effect of general self-efficacy was significant (the mediating effect value was 0.04); The chain mediating effect between interpersonal distress and general self-efficacy was significant (the mediating effect value was 0.01). Therefore, hypothesis 2, hypothesis 3, and Hypothesis 4 are valid.

**TABLE 7:** Bootstrap analysis of the significance test of mediation effect.

Effect path	Standardized indirect effect estimation	95% Confidence interval		Effect
		Upper limit	Lower limit	
Empowering motivational climate in physical education → interpersonal disturbance → social responsibility	0.014	0.012	0.023	0.048
Empowering motivational climate in physical education → general self-efficacy → social responsibility	0.043	0.047	0.092	0.148
Empowering motivational climate in physical education → interpersonal disturbance → general self-efficacy → social responsibility	0.009	0.008	0.021	0.031

*N = 802.*

* p < 0. 05; ** p < 0. 01.

## Discussion

### The Relationship Between Empowering Motivational Climate in Physical Education and Social Responsibility

This study found that the empowering motivational climate in physical education can positively predict social responsibility, which is also supported by other researchers (Mouratidou et al., 2007; Pengyu and Jianing, 2020). In the physical education teaching with more autonomous motivation style, students can produce less self involvement and more learning interest in physical education (Goudas et al., 1994), which will produce a higher level of development of students’ sports spirit, social responsibility and prosocial behavior (Biddle et al., 2003). Students’ independent support from teachers in sports activities promotes their own more self-determined behaviors, which plays an important role in cultivating students’ willingness to physical activities and behavioral norms for independent exercise (Lim and Wang, 2009). In the context of empowerment motivation climate, school physical education is more beneficial to the development of moral responsibility of middle school students, while in the context of empowerment motivation climate, the development of moral responsibility of school physical education needs to be further confirmed (Pengyu and Jianing, 2020).

### The Mediating Role of Interpersonal Disturbance and General Self-Efficacy

This study found that interpersonal disturbance plays an intermediary role between the empowering motivational climate in physical education and social responsibility, which shows that interpersonal distress is the key factor to understand the relationship between the empowering motivational climate in physical education and social responsibility. The empowering motivational climate in physical education can satisfy students’ sense of relationship, promote the good development of interpersonal relationship and reduce the level of interpersonal disturbance. This result is the same as that of previous studies (Ntoumanis, 2001). Interpersonal disturbance can negatively predict the social responsibility of high school students, which is consistent with the results of previous studies (Huang et al., 2016). In physical education teaching, the teacher-created empowering motivational climate can enable students to obtain sufficient interpersonal support, promote the mutual communication and emotional connection between students, obtain good interpersonal relationship, and reduce interpersonal disturbance. Research shows that social responsibility is based on the emotion of people’s relationship as an important condition or criterion. The closer the emotion is, the deeper the friendship is, the stronger the sense of responsibility is (Huang et al., 2016). Therefore, reducing interpersonal disturbance and improving interpersonal relationship between individuals and others are conducive to improving individuals’ awareness and behavior of taking responsibility for others, so as to Wray-Lake their social responsibility. In reality, many high school students are faced with interpersonal disturbance, therefore, we should pay attention to the impact of interpersonal relationship on high school students’ ideology and morality, especially social responsibility, to help high school students correctly understand and deal with their own interpersonal relationship.

### The Mediating Role of General Self-Efficacy

This study shows that, in addition to interpersonal disturbance, general self-efficacy plays a partial intermediary role between the empowering motivational climate in physical education and social responsibility, that is, the empowering motivational climate in physical education can not only directly affect high school students’ sense of social responsibility, but also affect high school students’ sense of social responsibility by affecting general self-efficacy. This result is also supported by other researchers (Yingqi, 2018). In the existing research on the empowering motivational climate in physical education and general self-efficacy, researchers found that the empowering motivational climate in physical education has a significant positive effect on general self-efficacy (Yue, 2012). Teachers with empowering motivational climate style will encourage students to try new sports skills in the process of physical education teaching, sincerely understand the uniqueness of each student, appreciate and care for students, and give students positive evaluation, trust, and support. In this process, students’ self-confidence and self-efficacy in learning will be improved. Individuals with high self-efficacy are more inclined to perform prosocial behaviors. Both prosocial behaviors and social responsibility are effective predicators of altruistic behaviors, so they are more conducive to the cultivation of responsibility (Yu, 2015).

### The Chain Mediating Effect of Interpersonal Disturbance and General Self-Efficacy

This study further found that the empowering motivational climate in physical education could predict high school students’ social responsibility through the chain mediating effect of interpersonal disturbance and general self-efficacy. According to the theory of Differential Responsibility Consciousness of Chinese people proposed by Ming and Jinang (2010), people will measure their own responsibilities according to how close they are to others. In physical education, teachers create a positive empowering motivational climate environment, which is conducive to improving the relationship between students and teachers, and reducing the negative effects brought by interpersonal disturbance. Such a good emotional relationship improves their awareness and behavior of taking responsibility for others. This result can also be explained from Social Cognitive Theory. Self-efficacy refers to the confidence that one is able to complete a task. The higher the level of self-efficacy, the higher the confidence to complete the task, and the greater the possibility of completing the task. The support and encouragement provided by teachers in physical education further enhance students’ self-confidence in completing teaching tasks. Self-efficacy and social responsibility belong to positive behavior and attitude tendencies. Self-efficacy is significantly related to teenagers’ social responsibility. Individuals with high self-efficacy are more likely to adopt positive coping styles, try their best to bring benefits to others or society (Hong et al., 2015).

Therefore, the empowering motivational climate in physical education can provide high school students with an environment for interpersonal communication and reduce interpersonal distress, which is negatively correlated with general self-efficacy (Yanyan et al., 2015). Therefore, it can be proved that the chain mediation between interpersonal disturbance and general self-efficacy is feasible, and it can play a partial intermediary role between the empowering motivational climate in physical education and social responsibility. To a certain extent, the intermediary effect model reveals the internal mechanism of empowering motivational climate in physical education to improve social responsibility, which has a certain guiding value for the work practice of improving the social responsibility of high school students. Therefore, for moral educators in high school, on the premise of giving full play to the important role of physical education teachers’ empowering motivational climate, they can effectively improve high school students’ social responsibility by reducing students’ interpersonal disturbance and improving general self-efficacy.

## Limitations and Future Directions

This study discusses the relationship between the empowering motivational climate in physical education and social responsibility, constructs a chain intermediary model, and reveals the internal mechanism of the influence of the empowering motivational climate in physical education on social responsibility, which has important theoretical value for understanding the causes of high school students’ social responsibility, At the same time, it also provides a preliminary basis for studying the causal relationship between the variables. However, the results of this study are still limited to the correlation between variables, and the causal relationship between variables cannot be inferred. In the future, longitudinal tracking experimental intervention research design can be used to more effectively explain the empowering motivational climate in physical education on high school students’ social responsibility. In addition, this study only considered the mediating effect of interpersonal disturbance and general self-efficacy, but in fact there may be other mediating variables such as physical education learning interest, rumination, emotional intelligence and so on, which need to be further explored. In future research, it is necessary to highlight the fundamental task of “Building Morality and Cultivating People” in the new era, adhere to the educational concept of “health first,” based on the all-round development of teenagers’ physical and mental health, and follow the fundamental requirements of “helping students enjoy fun in physical exercise, enhance their physique and improve their personality,” deeply study the dimensions of motivational climate (such as empowering and disempowering motivational climate) and the impact of relevant variables.

## Conclusion

The empowering motivational climate in physical education can not only positively predict the social responsibility of high school students, but also indirectly predict the social responsibility through the independent intermediary role of interpersonal disturbance and general self-efficacy, as well as the chain intermediary role of interpersonal disturbance and general self-efficacy.

## Data Availability Statement

The original contributions presented in the study are included in the article/Supplementary Material, further inquiries can be directed to the corresponding author.

## Ethics Statement

The studies involving human participants were reviewed and approved by the Institutional Review Board of the School of Physical Education and Health at Zhaoqing University of China. The participants provided their written informed consent to participate in the study.

## Author Contributions

K-LG and ZH: conceptualization and investigation. Q-SM: methodology. S-JY: software. Q-SM and CL: validation. S-JY and ZH: formal analysis. CL and K-LG: resources and supervision. S-JY and Q-SM: data curation, writing—original draft preparation, and visualization. K-LG and Q-SM: writing—review and editing. K-LG: project administration and funding acquisition. All authors have read and agreed to the published version of the manuscript.

## Conflict of Interest

The authors declare that the research was conducted in the absence of any commercial or financial relationships that could be construed as a potential conflict of interest.

## Publisher’s Note

All claims expressed in this article are solely those of the authors and do not necessarily represent those of their affiliated organizations, or those of the publisher, the editors and the reviewers. Any product that may be evaluated in this article, or claim that may be made by its manufacturer, is not guaranteed or endorsed by the publisher.

## References

[B1] Ames C. (1992). “Achievement goals, motivational climate, and motivational processes,” in *Motivation In Sport And Exercise*, ed. Roberts G. C. (Champaign: Human Kinetics), 161–176.

[B2] Appleton P. Ntoumanis N. Quested E. Viladrich C. Duda J. L. (2016). Initial validation of the coach-created Empowering and Disempowering Motivational Climate Questionnaire (EDMCQ-C). *Psychol. Sport Exerc.* 22 53–65. 10.1016/j.psychsport.2015.05.008

[B3] Bandura A. (1999). “A social cognitive theory of personality,” in *Handbook of Personality 2nd ed*, eds Pervin L. John O. (New York: Guilford Publications), 154–196.

[B4] Baoling H. Jiliang D. (2015). An empirical study on the social responsibility structure of post-90s college students. *Contin. Educ. Res.* 6 110–112.

[B5] Biddle S. Wang C. K. J. Kavussanu M. Spray C. (2003). Correlates of achievement goal orientations in physical activity:A systematic review of research. *Eur. J. Sport Sci.* 3 1–20. 10.1080/17461390300073504

[B6] Braithwaite R. Spray C. M. Warburton V. E. (2011). Motivational climate interventions in physical education:A meta-analysis. *Psychol. Sport Exerc.* 12 628–638. 10.1016/j.psychsport.2011.06.005

[B7] Caikang W. Zhongfeng H. Yong L. (2001). Evidences for reliability and validity of the Chinese version of General Self-Efficacy Scale. *Chin. J. Appl. Psychol.* 7 37–40.

[B8] Deci E. L. Ryan R. M. (1985). *Intrinsic Motivation And Self-Determination In Human Behavior.* New York: Springer Science & Business Media.

[B9] Deci E. L. Ryan R. M. (2000). The “What” and “Why” of Goal Pursuits: human Needs and the Self-Determination of Behavior. *Psychol. Inq.* 11 227–268. 10.1207/S15327965PLI1104_01

[B10] Demaray M. K. Malecki C. K. (2002). Critical levels of perceived social support associated with student adjustment. *Sch. Psychol. Q.* 17 213–241. 10.1521/scpq.17.3.213.20883

[B11] Dingding W. Yi J. Zhao K. Shen T. (2018). Relationship between physical self-esteem and general self-efficacy in undergraduate students:mediating effect of interpersonal relationship. *J. Guizhou Norm. Univ.* 36 111–117. 10.16614/j.cnki.issn1004-5570.2018.01.017

[B12] Duda J. L. (2001). “Achievement goal research in sport: Pushing the boundaries and clarifying some misunderstandings,” in *Advances In Motivation In Sport And Exercise*, ed. Roberts G. C. (Leeds: Human Kinetics), 129–182.

[B13] Duda J. L. (2013). The conceptual and empirical foundations of Empowering Coaching™:Setting the stage for the PAPA project. *Int. J. Sport Exerc. Psychol.* 11 311–318. 10.1080/1612197X.2013.839414 27055568

[B14] González-Valero G. Ubago-Jiménez J. L. Ramírez-Granizo I. A. Puertas-Molero P. (2019). Association between motivational climate, adherence to mediterranean diet, and levels of physical activity in physical education students. *Behav. Sci.* 9:37. 10.3390/bs9040037 PMC6523412 30979088

[B15] Goudas M. Biddle S. Fox K. (1994). Achievement Goal Orientations and Intrinsic Motivation in Physical Fitness Testing with Children. *Pediatr. Exerc. Sci.* 6 159–167. 10.1123/pes.6.2.159

[B16] Guixue Y. (2013). *The Effect of Teacher-Student’ Speech Interaction on Stufent’s Self-Efficacy in the Classroom.* Ph.D. thesis. Jinhua: Zhejiang Normal University.

[B17] Hao Z. (2011). Learning self-efficacy:cultivated in teacher evaluation and penetration. *Modern Educ. Sci.* 8 24–25. 10.13980/j.cnki.xdjykx.gjyj.2011.08.024

[B18] Hao Z. Lirong L. (2004). Statistical Remedies for Common Method Biases. *Adv. Psychol. Sci.* 12 942–950.

[B19] Hellison D. Wright P. (2003). Retention in an urban extended day program: a process-based assesment. *J. Teach. Phys. Educ.* 22 369–381. 10.1123/jtpe.22.4.369

[B20] Hong L. Mingjun Z. Nan Y. Lin C. Hui S. Zhendong Z. (2015). Relationship between generalself-eficacy and coping styleam ong college students. *Chin. J. Public Health* 31 1202–1204.

[B21] Hongmei S. Shanyan Y. Zhihong H. Yong L. (2018). The Influence of social support on social responsibility in medical students: the Mediating role of self-efficacy. *China Higher Med. Edu.* 3 29–30.

[B22] Huang S. Han M. Zhang M. (2016). The impact of interpersonal relationship on social responsibility. *Acta Psychol. Sin.* 48 578–587. 10.3724/SP.J.1041.2016.00578

[B23] Jianwen C. Tao W. (2007). An Analysis of the Relation between Self-Esteem and Self-efficacy. *Adv. Psychol. Sci.* 15 624–630.

[B24] Jianyu Z. Xiaohua H. (2016). Discussing the relationship among regulatory emotional self-efficacy, Interpersonal disturbance andsubjective well-being. *J. Gannan Norm. Univ.* 37 110–113. 10.13698/j.cnki.cn36-1037/c.2016.02.029

[B25] Jie F. MinQiang M. (2012). Assessing point and interval estimation for the mediating effect:distribution of the product, nonparametric Bootstrap and markov chain monte carlo methods. *Acta Psychol. Sin.* 44 1408–1420. 10.3724/SP.J.1041.2012.01408

[B26] Jie F. Minqiang Z. Hawjeng C. (2012). Mediation analysis and effect size measurement:retrospect and prospect. *Psychol. Dev. Educ.* 28 105–111. 10.16187/j.cnki.issn1001-4918.2012.01.015

[B27] Kolovelonis A. Keramidas P. Krommidas C. Goudas M. (2015). The relationship between motivational climate and social behavior in physical education. *J. Phys. Act. Nutr. Rehabil.* 1 1–11.

[B28] Lan S. Qizong H. Chun S. (2015). Review on the research of sense of social responsibility of Chinese adolescent in last ten years. *Educ. Res. Month.* 11 22–28. 10.16477/j.cnki.issn1674-2311.2015.11.004

[B29] Lim B. S. C. Wang C. K. J. (2009). Perceived autonomy support, behavioural regulations in physical education and physical activity intention. *Psychol. Sport Exerc.* 10 52–60. 10.1016/j.psychsport.2008.06.003

[B30] Liwei Z. Zhixiong M. (2019). “Eight problems worthy of continuous exploration by sports psychologists,” in *Summary of theses of the 11th National Sports Science Conference*, (Beijing: China Sports Science Society).

[B31] Manzano-Sánchez D. Gómez-Mármol A. Valero-Valenzuela A. Jiménez-Parra J. F. (2021). School climate and responsibility as predictors of antisocial and prosocial behaviors and violence: a study towards self-determination theory. *Behav. Sci.* 11:36. 10.3390/bs11030036 33802667 PMC8002525

[B32] Metzler M. (2017). *Instructional Models In Physical Education.* New York: Routledge.

[B33] Milton D. Appleton P. R. Bryant A. Duda J. L. (2018). Initial Validation of the Teacher-Created Empowering and Disempowering Motivational Climate Questionnaire in Physical Education. *J. Teach. Phys. Educ.* 37 340–351. 10.1123/jtpe.2018-0119

[B34] Ming L. Jinang G. (2010). A Socio-cultural psychological analysis of responsibility. *J. Nanjing Norm. Univ.* 3 111–115.

[B35] Mouratidou K. Goutza S. Chatzopoulos D. (2007). Physical education and moral development: an intervention programme to promote moral reasoning through physical education in high school students. *Eur. Phys. Educ. Rev.* 13 41–56. 10.1177/1356336x07072675

[B36] Nicholls J. G. (1989). *The Competitive Ethos And Democratic Education.* Cambridge: Harvard University Press.

[B37] Ning C. Chaoyi H. (2015). The developmental characteristics of contemporary Adolescents’ responsibility in China:based on a survey of six provinces and cities. *Chin. Youth Stud.* 88 56–59. 10.19633/j.cnki.11-2579/d.2015.12.009

[B38] Ntoumanis N. (2001). A self-determination approach to the understanding of motivation in physical education. *Br. J. Educ. Psychol.* 71 225–242. 10.1348/000709901158497 11449934

[B39] Pengyu W. Jianing L. (2020). Prediction of personal and social responsibility by psychological environment in physical education:Mediating effect of basic psychological needs. *J. Shenyang Sport Univ.* 39 30–36. 10.12163/j.ssu.20200430

[B40] Podsakoff P. M. MacKenzie S. B. Lee J.-Y. Podsakoff N. P. (2003). Common method biases in behavioral research:A critical review of the literature and recommended remedies. *J. Appl. Psychol.* 88 879–903. 10.1037/0021-9010.88.5.879 14516251

[B41] Qian Z. Shou-cai G. (2009). Research of Troubled Student Interpersonal Relationship, Student General Self-efficacy and TheirCorrelation. *J. Yangzhou Univ.* 13 36–40. 10.19411/j.cnki.1007-8606.2009.04.010

[B42] Qiang C. (2011). Different Effects of the Family Function and Quality of Life on the Social Responsibility among Ordinary High School and Vocational High School Students. *Chin. J. Appl. Psychol.* 17 145–152.

[B43] Richang Z. (1999). *Psychological Diagnosis Of College Students.* Jinan: Shandong Education Press, 330–344.

[B44] Rui G. Xuerong L. Jianying L. Jiancheng L. Shusong D. (2017). Exploring the mediating effect and moderating effect of self-efficacy between coping style and sensation seeking among adolescents. *Modern Prev. Med.* 44 1080–1082.

[B45] Ruimiao H. (2012). *study On The Relationship Between Social Support Andsocial Responsibility Of Junior Middle School Students.* Ph.D. thesis. Guangzhou: Guangzhou University.

[B46] Schwarzer R. Mueller J. Greenglass E. (1999). Assessment of perceived general self-efficacy on the internet:Data collection in cyberspace. *Anxiety Stress Coping* 12 145–161. 10.1080/10615809908248327

[B47] Van den Berghe L. Vansteenkiste M. Cardon G. Kirk D. Haerens L. (2014). Research on self-determination in physical education:Key fifindings and proposals for future research. *Phys. Educ. Sport Pedagogy* 19 97–121. 10.1080/17408989.2012.732563

[B48] Wenfeng H. Fuming X. (2004). Relationship of college students’ learning adaptability with general self-efficacy and social support. *Chin. J. Clin. Psychol.* 4 369–370. 10.16128/j.cnki.1005-3611.2004.04.015

[B49] Wray-Lake L. Syvertsen A. K. (2011). The developmental roots of social responsibility in childhood and adolescence. *New Dir. Child Adolesc. Dev.* 134 11–25. 10.1002/cd.308 22147598

[B50] Xue L. (2004). *A Study On The Social Responsibility Structure And Its Developmental Characteristics Of Middle School Students.* Ph.D. thesis. Chong qing: Southwest Normal University.

[B51] Yanyan L. Jie Z. Xin Z. (2015). A Study on the Relationship between Dormitory Interpersonal relationship and General Self-efficacy of College Students. *China Adult Educ.* 16 118–120.

[B52] Yawen Z. Fangfang P. Jinbing A. Ruiyuan G. (2020). Relationship between empathy and interpersonal disturbance and its gender difference among middle school students. *Chin. Ment. Health J.* 34 337–341.

[B53] Yingqi Y. (2018). *The Impact Of Teachers’ Transformational Leadership Style On Junior High School Students’ Responsibility:The Mediating Role Of Self-Efficacy.* Ph.D. thesis. Changsha: Hunan Normal University.

[B54] Yong L. Xiaohong T. (2008). A study on the structure and characteristics of the middle school students’ social responsibility. *Chin. J. Spec. Educ.* 5 78–82. 10.1006/ceps.1998.0978 9878206

[B55] Yongzhi J. Xiaoli B. (2014). On the relationship between college students’mobile phone addiction and loneliness: the Intermediary role of online social support. *Chin. J. Spec. Educ.* 1 41–47.

[B56] Yu J. (2015). *Study on Relationship Between Regulatory Emotional Self-Efficacy, Perceived Self-Efficacy to Manage Interpersonal Relationship and Prosocial Behaviors.* Ph.D. thesis. Jinan: Jinan University.

[B57] Yue Y. (2012). *The Influence of Teaching Style on Student’s Achievementahe Intermediary Role of Academic Self-Efficacy, Academic Interest.* Ph.D. thesis. Shanghai: Shanghai Normal University.

[B58] Zhonglin W. Binbin H. Dandan T. (2018). Preliminary Work for Modeling Questionnaire Data. *J. Psychol. Sci.* 41 204–210. 10.16719/j.cnki.1671-6981.20180130

